# Unsaturated Fatty Acids Revert Diet-Induced Hypothalamic Inflammation in Obesity

**DOI:** 10.1371/journal.pone.0030571

**Published:** 2012-01-18

**Authors:** Dennys E. Cintra, Eduardo R. Ropelle, Juliana C. Moraes, José R. Pauli, Joseane Morari, Claudio T. de Souza, Renato Grimaldi, Marcela Stahl, José B. Carvalheira, Mario J. Saad, Licio A. Velloso

**Affiliations:** 1 Laboratory of Cell Signaling, University of Campinas, Campinas, Brazil; 2 Department of Internal Medicine, University of Campinas, Campinas, Brazil; 3 Faculty of Applied Sciences, University of Campinas, Campinas, Brazil; 4 Faculty of Food Engineering, University of Campinas, Campinas, Brazil; Instituto de Química - Universidade de São Paulo, Brazil

## Abstract

**Background:**

In experimental models, hypothalamic inflammation is an early and determining factor in the installation and progression of obesity. Pharmacological and gene-based approaches have proven efficient in restraining inflammation and correcting the obese phenotypes. However, the role of nutrients in the modulation of hypothalamic inflammation is unknown.

**Methodology/Principal Findings:**

Here we show that, in a mouse model of diet-induced obesity, partial substitution of the fatty acid component of the diet by flax seed oil (rich in C18:3) or olive oil (rich in C18:1) corrects hypothalamic inflammation, hypothalamic and whole body insulin resistance, and body adiposity. In addition, upon icv injection in obese rats, both ω3 and ω9 pure fatty acids reduce spontaneous food intake and body mass gain. These effects are accompanied by the reversal of functional and molecular hypothalamic resistance to leptin/insulin and increased POMC and CART expressions. In addition, both, ω3 and ω9 fatty acids inhibit the AMPK/ACC pathway and increase CPT1 and SCD1 expression in the hypothalamus. Finally, acute hypothalamic injection of ω3 and ω9 fatty acids activate signal transduction through the recently identified GPR120 unsaturated fatty acid receptor.

**Conclusions/Significance:**

Unsaturated fatty acids can act either as nutrients or directly in the hypothalamus, reverting diet-induced inflammation and reducing body adiposity. These data show that, in addition to pharmacological and genetic approaches, nutrients can also be attractive candidates for controlling hypothalamic inflammation in obesity.

## Introduction

Defective hypothalamic activity plays an important role in the development of obesity [1], [2], [3]. A number of recent studies have shown that in both diet-induced and genetically-determined animal models of obesity, inflammation of the hypothalamus is an important mechanism leading to the anomalous control of caloric intake and energy expenditure [4], [5], [6], [7], [8], [9]. Saturated fatty acids, highly consumed in western diets, induce hypothalamic inflammation by activating signal transduction though TLR4, which leads to endoplasmic reticulum stress, *in situ* expression of inflammatory cytokines and eventually, apoptosis of neurons, all contributing to the development of adipostatic hormone resistance and anomalous expression of the neurotransmitters involved in the regulation of energy homeostasis [5], [6].

Both genetic and pharmacological approaches, aimed at restraining hypothalamic inflammation, have proven useful for reducing hypothalamic dysfunction, correcting resistance to leptin and insulin and reducing body mass. In this context, several proteins involved in the inflammatory response in the hypothamus have been targeted with generally positive outcomes. Some examples include SOCS3 and IKK [8], [10], which have been targeted by gene-based approaches, and TNF-α, JNK and TLR4, which have been targeted by pharmacological means [4], [5], [11]. Although these results unveil promising approaches for the treatment of obesity, the known pleotropy of all these inflammatory pathways, and the need to concentrate the effect on a limited region of the brain, impose a certain dose of uncertainty regarding the future development of anti-inflammatory drugs to tackle obesity.

In other tissues and cell types, unsaturated fatty acids have well known anti-inflammatory effects, which range from the inhibition of the lipoxygenase and cycloxigenase pathways and decrease of neutrophil adhesion [12] to the reduction of inflammatory cytokine expression [13] and inhibition of TLR4 signaling [14]. Since nutritional approaches are the basis for all prophylactic and therapeutic protocols employed for dealing with obesity, we decided to evaluate the effects of two unsaturated fatty acids on hypothalamic inflammation in obesity. Here, we show that, acting either as nutrients or directly in the hypothalamus, linolenic (C18:3, ω3) and oleic (C18:1, ω9) unsaturated fatty acids have outstanding anti-inflammatory effects, correcting hypothalamic dysfunction and reducing body mass.

## Materials and Methods

### Experimental animals

Rats and mice were obtained from the University of Campinas Breeding Center. Male Wistar rats (*Rattus norvegicus*) with an initial body mass of 180 g, were allowed access to standard rodent chow (CT) or high-fat diet (HF,) according to a protocol described below and in Table 1. Male Swiss mice (*Mus musculus*) with an initial body mass of 13 g, were allowed access to standard rodent chow (CT), high-fat diet (HF), HF + flax seed oil (FS) (stepwise substitutions corresponding to 10, 20 or 30% of total caloric value) or HF + olive oil (OL) (stepwise substitutions corresponding to 10, 20 or 30% of total caloric value), as described below and in Table 1. All animals received diet and water *ad libitum* and were maintained in individual cages at 21±2°C, with a 12-h dark/12-h light cycle. All experiments were conducted in accordance with the principles and procedures described by the National Institutes of Health Guidelines for the Care and Use of Experimental Animals and were approved by the University of Campinas Ethical Committee (ID 2010/0256).

**Table 1:** Macronutrient composition of experimental diets (g/kg).

Ingredients	CT	HF	FS (10%)	FS (20%)	FS (30%)	OL (10%)	OL (20%)	OL (30%)
Corn Starch	427.5	115.5	115.5	115.5	115.5	115.5	115.5	115.5
Casein	200	200	200	200	200	200	200	200
Sucrose	132	132	132	132	132	132	132	132
Dextrinated Starch	100	100	100	100	100	100	100	100
Soybean Oil	40	40	40	40	40	40	40	40
Lard	0	312	208	104	0	208	104	0
Flax Seed Oil	0	0	104	208	312	0	0	0
Olive Oil	0	0	0	0	0	104	208	312
Cellulose	50	50	50	50	50	50	50	50
Mineral Mix	35	35	35	35	35	35	35	35
Vitamin Mix	10	10	10	10	10	10	10	10
L-Cysteine	3	3	3	3	3	3	3	3
Choline	2.5	2.5	2.5	2.5	2.5	2.5	2.5	2.5
Total	1000	1000	1000	1000	1000	1000	1000	1000

CT, control diet; HF, high fat diet; FS, flax seed oil substituted; OL, olive oil substituted.

### Experimental protocols

**Figure 1:**
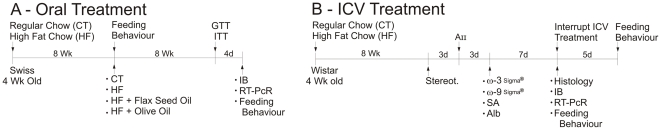
Experimental protocols. A, Four-week old male Swiss mice were fed regular chow (CT) or high-fat diet (HF) for eight weeks and then randomly divided into eight groups: CT mice, maintained for another eight weeks on the same CT diet; or HF mice which were either maintained on the same HF diet; or transferred to diets containing substitution of lard by olive or flax seed oils to final concentrations of 10, 20 or 30%, according to Table 1. At the end of the experimental period, the mice were submitted to a glucose tolerance test (GTT) and an insulin tolerance test (ITT), four days later, feeding behavior was evaluated and samples were obtained for immunobloting (IB) and real-time PCR (RT-PCR). B, Four-week old male Wistar rats were fed for eight weeks on CT or HF diets and then submitted to intracerebroventricular (icv) cannulation. Drinking response elicited by angiotensin II (AII) was tested three days after cannulation and responsive rats were treated icv with either ω3, ω9, stearic acid (SA) or diluents (Alb) for seven days. Feeding behavior and body mass were determined throughout the experimental period. At the end of the experimental period some rats were randomly selected for histology, IB and RT-PCR; the remainder of the rats was followed up for feeding behavior for an additional five days after the discontinuation of icv treatment.

Experimental animals were submitted to two distinct approaches to evaluate the role of unsaturated fatty acids in hypothalamic dysfunction. Swiss mice (Fig. 1A), fed for 8 weeks on a HF diet, were randomly assigned to one of the following regimens: maintained for another 8 weeks on HF diet; introduced to a FS fat substitution diet of 10, 20 or 30%, according to Table 1 or introduced to a OL fat substitution diet of 10, 20 or 30%, according to Table 1. The FS and OL source oils were always maintained in amber bottles and stored at 22°C. The diets were prepared every ten days and kept protected from light, at 4°C. At the end of the experimental period, mice were submitted to glucose and insulin tolerance tests (GTT and ITT, respectively), as described below. Finally, after four days, feeding behavior was evaluated and samples were collected for real-time PCR and immunoblot. In a separate experiment, diet preference was tested in lean male Swiss mice, which were fasted for 10h and then exposed simultaneously to two recipients containing equal amounts of two different diets. The amount of each diet consumed over a period of 12h was recorded and the caloric value was calculated. Results are presented as the relative preference for one of the diets as obtained from the ratio of caloric intake from each diet. In the second approach (Fig. 1B), Wistar rats were fed on a HF diet for 8 weeks and then were submitted to icv cannulation. After three days, the efficiency of the cannulation was tested with angiotensin II, as described below and, after another three days the rats were randomly divided to receive either albumin (2 µl, 75 µM), ω3 (2 µl, 225 µM), ω9 (2 µl, 225 µM) or stearic acid (2 µl, 225 µM) icv for seven days. At the end of the experimental period some rats were selected for evaluation of feeding behavior and collection of samples for real-time PCR, histology and immunoblotting. The remainder of the animals was selected for evaluation of feeding behavior for up to five days after interruption of the icv treatments. For GPR120 localization and signal transduction studies, 8 weeks lean rats were employed.

### Antibodies, chemicals and buffers

The reagents for SDS–polyacrylamide gel electrophoresis and immunoblotting were from Bio-Rad (Richmond, CA, USA). HEPES, phenylmethylsulfonyl fluoride, aprotinin, dithiothreitol, Triton X-100, Tween 20, glycerol, oleic acid/C18:1/ω9 (O-1383–1G), linolenic acid/C18:3/ω3 (L-2376), stearic acid/C18:0 (L-5376), bovine serum albumin (fraction V) and bovine serum albumin fatty acid free (A-6003), were purchased from Sigma-Aldrich (St. Louis, MO, USA). The reagents for chemoluminescence labeling of proteins in blots were from Amersham (Aylesbury, UK). Human recombinant insulin (Humulin R) and sodium thiopental were from Lilly (Indianapolis, IN, USA). Anti-Akt (sc-1618, goat polyclonal), anti-phospho [Ser473] Akt (sc-7985-R, rabbit polyclonal), anti-FOXO1 (sc-11350, rabbit polyclonal), anti-phospho [Ser256] FOXO1 (sc-22158-R, rabbit polyclonal), anti-phospho [Tyr^1007/1008^] JAK2 (rabbit polyclonal),anti-JAK2 (sc-278, rabbit polyclonal), anti-STAT3 (sc-483, rabbit polyclonal), anti-TNF-α (sc-1350, goat polyclonal), anti-IL-6 (sc-1265, goat polyclonal), anti-IL-10 (sc-1783, goat polyclonal), anti-F4/80 (sc-25830, rabbit polyclonal), anti- inducible nitric oxide synthase (iNOS), anti-phospho [Ser 32] IκB-α (sc-7977, rabbit polyclonal), anti-IκB-α (sc-1643, mouse monoclonal), anti-phospho [Thr 183/185] c-Jun N terminal kinase (JNK) (sc-12882, rabbit polyclonal), anti-JNK (sc-1648, mouse monoclonal), anti-stearoil CoA desaturase-1 (SCD-1) (sc-23016, rabbit polyclonal), anti-carnitine palmitoyl transferase 1 (CPT1) (sc-20514, goat polyclonal), anti-fatty acid synthase (FAS) (sc-16146, goat polyclonal), anti-ACC (#3662, rabbit polyclonal), anti-uncoupled protein-1 (UCP-1) (sc-6529, goat polyclonal), anti-Bcl2 (sc-783, rabbit polyclonal), anti-Bax (sc-6236, rabbit polyclonal), anti-caspase 3 (sc-7148, rabbit polyclonal), anti-NPY (sc-14727, goat polyclonal), anti-GPR120 (sc-99105, rabbit polyclonal), FITC-conjugated mice anti-rabbit and rhodamine-conjugated anti-goat antibodies were from Santa Cruz Biotechnology (Santa Cruz, CA, USA). Anti-phospho [Tyr705] STAT3 (rabbit polyclonal, #9135), anti-TAB1 (#3225, rabbit polyclonal), anti-TAK1 (#5206, rabbit polyclonal), anti-β-arrestin 2 (#3857, rabbit polyclonal) and anti-SOCS3 (#2923, rabbit polyclonal) were from Cell Signaling (Danvers, MA, USA). Anti-phospho [Ser^79^] acetyl CoA carboxylase (ACC) (#07-184, rabbit polyclonal) was from Upstate Biotechnology (Charlottesville, VA, USA). Anti β-actin (#ab6276, mouse monoclonal) was from Abcam (Cambridge, MA). All the chemicals used in the real-time PCR experiments and 4,6-diamidino-2-phenylindole dihydrochloride (DAPI) used in immunofluorescence staining were purchased from Invitrogen and Applied Biosystems.

### Intraperitoneal insulin tolerance test (ipITT)

After 6 h of fasting, CT, HF, FS- and OL-substituted mice groups were submitted to an insulin tolerance test (1 U/kg body weight^−1^ of insulin). Mice were injected with insulin and blood samples were collected at 0, 4, 8, 12 and 16 min from the tail vein for serum glucose determination. The constant for the rate of serum glucose disappearance was calculated using the formula 0.693/biological half-life (*t*1*/*2). The plasma glucose *t*1*/*2 was calculated from the slope of last square analysis of the plasma glucose concentration during the linear phase of decline [15].

### Intraperitoneal glucose tolerance test (ipGTT)

After 6 h of fasting, mice were submitted to a glucose tolerance test. After collection of an unchallenged sample (time 0), a solution of 20% glucose (2.0 g/kg body weight) was administered into the peritoneal cavity. Blood samples were collected from the tail vein at 30, 60, 90 and 120 min for determination of glucose concentrations. Results are presented as the area under glucose curves. Serum glucose of both ipITT or ipGTT were determined using a glucose meter (Roche Diagnostic, Rotkreuze, Switzerland).

### Feeding behaviour

Rats deprived of food for 6 h with free access to water were icv injected (2μl) with solutions containing albumin (75 µM) or leptin (1.0 µM). Thereafter, diets were reintroduced and food intake was determined at the end of a 12-h period.

### Icv cannulation

For icv cannulation, Wistar rats were used after 8 weeks of treatment with CT or HF diets. The rats were stereotaxically instrumented under sodium thiopental [15 mg (kg body weight)^−1^] anesthesia using a Stoelting stereotaxic apparatus, according to a previously described method [5]. Cannula efficiency was tested 3 d after cannulation by the evaluation of the drinking response elicited by icv angiotensin II [16]. Stereotaxic coordinates were anteroposterior, 0.2 mm/lateral, 1.5 mm/depth, 4.0 mm. Icv-cannulated rats were treated with 2 µl of each fatty acid for 7 d. Controls were treated with a similar volume of albumin. Fatty acids for icv injection were always diluted in ultrapure water containing HBP detergent (0.1%) and fatty acid free BSA (75 µM). The volumes injected were always 2 µl/dose. The final concentration of fatty acids was always 225 µM. In some experiments, rats were treated icv with pure oleic, linolenic or stearic acids (225 µM). LPS contamination of the fatty acids, HBP and BSA preparations were evaluated by Limulus amoebocyte lysate assay produced by Associates of Cape Cod. LPS in the diets were evaluated by HPLC as described below. Only trace amounts of LPS (ranging from 0.030 to 0.070 EU/nmol) were detected in the reagents. According to a previously study [17], these levels of LPS do not interfere with the results.

### HPLC

Determination of general lipid content and LPS in diets was performed using a HPLC method described previously [18]. Briefly, fatty acids were derivatized with 4-bromomethyl-7-coumarin and the analysis performed in a Shimadzu model LC-10A liquid chromatographer. The samples were eluted using a C8 column (25 cm × 4.6 id, 5 µm of particles) with a C8 precolumn (2.5 cm × 4.6 id, 5 µm of particles), 1.0 ml/min of acetonitrile/water (77%/23%, v/v) flow and fluorescence detector (325 nm excitation and 395 nm emission). For quantification of fatty acids, the capacity factor, elution sequence, linearity, recovery, precision, interference, and limit of detection were determined. The lower limit of detection was 1.0 pg and highly purified LPS was used as a tracer.

### Gas chromatography

The relative amounts of fatty acids in the diets, blood and hypothalami were determined by gas chromatography, using a Shimadzu Model 17A, under the following operating conditions: fused silica capillary column (100×0.25 mm; SP-2560); hydrogen as the carrier gas, with a flow rate of 20cm/s; oven temperature, initially 140°C for 5 min, was increased up to 240°C at 4°C/min and maintained at 240°Cfor 30 min; injector, split ratio was 1∶50, temperature 250°C; vaporization and detector temperatures were 250°Cand 260°C, respectively. The injection volume was 1 µl of sample solution. The retention times of methyl ester standards (Sigma Aldrich) were used to identify peaks. Lipids extracted from diets, blood and hypothalami were stored at −20°C protected from light.

### Immunohistochemistry and histology

Epididymal fat specimens were dehydrated with ethanol, cleared with xylene, and embedded in paraffin wax (Merck, São Paulo, Brazil). After inclusion, 4 mm sections were obtained on a microtome (CUT model 445, Olympus). Epididymal fat sections were stained with hematoxylin and eosin (Merck). Photomicrographs were obtained as described below. The mean area of adipocytes (average surface area of 30 randomly sorted adipocytes, per animal) was determined by the Imagelab Analysis software (Imagelab, Modena, Italy, v. 2.4). Hydrated, 4 µm sections of paraformaldehyde-fixed, paraffin embedded, central nervous system specimens were obtained from rats treated for 8 weeks with CT or HF diets plus the respective icv treatment (ω3 or ω9 injections). The expressions of F4/80, GPR120 and NPY were evaluated by indirect immunofluorescence staining, as described previously [19]. In short, hydrated sections were incubated for 30 min at room temperature with 2% rabbit pre-immune serum and then incubated for 12 h, at 4°C, in a moister chamber, with an anti-F4/80 (1∶50) or an anti-GRP120 (1∶100). Sections incubated with the anti-F4/80 antibody were then incubated with a FITC conjugated anti-rabbit IgG secondary antibody. Sections incubated with the anti-GRP120 antibody were then incubated with the second primary antibody against NPY (1∶50) for another 12 h, at 4°C, in the moister chamber. Finally, sections were incubated with FITC-, and rhodamine-conjugated secondary antibodies. Analysis and documentation of results were performed using a Leica FW 4500 B microscope. The hypothalami were sectioned from Bregma -2.1 to -2.3 mm. The anatomical correlations were made according to the landmarks given in a stereotaxic atlas [20]. The topographical views of the regions to be studied were obtained by hematoxylin-eosin staining of consecutive sections.

### Protein analysis by immunoprecipitation and immunoblotting

As soon as anaesthesia was assured by the loss of pedal and corneal reflexes, the skull was opened and the basal diencephalon, including the preoptic area and hypothalamus, was excised. The hypothalami were pooled and 125 µg of protein was used as a whole tissue extract. In some experiments, fragments of the brown adipose tissue (BAT) were used for whole protein extract preparation. For evaluating leptin or insulin responsive proteins, samples were obtained 15 min after leptin/insulin (2 µl, 1 µM solution of either leptin or insulin) injection, as shown for Kim and colleagues [21]. We carried out similar proceedings to extract the mice brains to collect the hypothlami. Tissues were pooled, minced coarsely and homogenized immediately in extraction buffer (1% Triton X-100, 100 mM Tris, pH 7.4, containing 100 mM sodium pyrophosphate, 100mM sodium fluoride, 10 mM EDTA, 10 mM sodium vanadate, 2 mM phenylmethanesulphonylfluoride (PMSF) and 0.1 mg ml^−1^ aprotinin) at 4°C with a Polytron PTA 20S generator (Brinkmann Instruments model PT 10/35) operated at maximum speed for 15 s. The extracts were centrifuged at 9,000 *g* and 4°C in a Beckman 70.1 Ti rotor (Palo Alto, CA, USA) for 45 min to remove insoluble material, and the supernatants of these tissues were used for protein quantification, using the Bradford method [22]. Proteins were denatured by boiling in Laemmli sample buffer. Samples containing 125 µg of protein extracts were separated by SDS–PAGE, transferred to nitrocellulose membranes and blotted with antibodies against TNF-α, IL-6, IL-10, iNOS, JNK, p-JNK, p-IκB-α, SOCS3, caspase3, BAX, Bcl2, p-ACC, ACC, FAS, CPT-1, SCD, UCP-1, JAK, p-JAK2, STAT3, p-STAT3, AKT, p-Akt, FoxO1, and β-actin. Specific bands were labeled by chemiluminescence and visualization was performed by exposure of the membranes to RX-films. For the evaluation of GPR120 signal transduction, rats were acutely treated with either ω3 or ω9 fatty acids (225 µM) and hypothalamic protein extracts were prepared. For each analysis, 500 µg protein was used for immunoprecipitation with GPR120, β-arrestin 2 or TAK1 antibodies. Immunoprecipitates were separated by SDS–PAGE and membranes were blotted with either β-arrestin or TAB1 antibodies.

*Real-time PCR.* Reverse-transcription was performed using total RNA from hypothalamic samples, as described previously [19]. NPY, POMC, CART and MCH mRNAs were measured in the hypothalami of rats treated with CD or HF diets and in intracerebroventricular cannulated rats treated with linolenic and oleic acid. Intron-skipping primers for NPY, AGRP, MCH and POMC were obtained from Applied Biosystems. Glyceraldehyde-3-phosphate dehydrogenase primers (Applied Biosystems) were used as a control. Real-time PCR analysis of gene expression was performed in an ABI Prism 7700 sequence detection system (Applied Biosystems). The optimal concentration of cDNA and primers, as well as the maximum efficiency of amplification, were obtained through five-point, two-fold dilution curve analysis for each gene. Each PCR contained 3.0 ng of reverse-transcribed RNA, 200 nM of each specific primer, SYBR SAFE PCR master mix, and RNase free water added to a 20 µl final volume. Real-time data were analyzed using the Sequence Detector System 1.7 (Applied Biosystems).

### Statistical analysis

Specific protein bands present in the blots were quantified by digital densitometry (ScionCorp). Analysis of variance was used to compare independent samples. Mean values ± SEM obtained from densitometry scans and from real-time PCR measurements, body mass determination, food intake, area under glucose curves and Kitt were compared using *Tukey's test* and *P<0.05* was accepted as statistically significant. In all experiments, we used five distinct animals from each of the experimental groups.

## Results

### Composition of the diets

HPLC and gas chromatography analysis of the diets showed that the high-fat diet (HF) contained 37% saturated fat, while chow contained 16% (Tables 1 and 2). The 10% substitution by FS led to an increase in the ω3 component of the diet from 1.3 to 15% while the 10% substitution by OL led to an increase in the ω9 component of the diet from 40 to 44% (Tables 1 and 2). No LPS contamination was detected in the diets as determined by HPLC.

**Table 2:** Fatty acid composition of the diets (% of total fat).

Fatty Acids	CT	HF	FS 10%	OL 10%
C14:0	0.64±0.25	1.19±0.05# a	0.04±0.01^b^	0.85±0.02^b^
C16:0	13.46±0.96	21.5±0.33# a	16.98±1.71^b^	18.88±0.06^b^
C18:0	2.24±0.57	11.13±0.42# a	8.72±0.87^b^	8.72±0.15^b^
C20:0	-	0.7±0.09^a^	0.21±0.01^b^	0.34±0.03^c^
Σ SFA	16.7±0.1	37.48±1.45# a	26.96±1.81^b^	31±1.68^b^
C16:1 ω7	-	2.02±0.14	1.4±0.09	1.76±0.13
C18:1 ω9	27.17±1.94	40.54±0.7# a	32.83±0.03^b^	44.56±0.8^a^
Σ MUFA	27.8±1.8	42.56±2.2# a	38.24±2.9^a^	49.19±3.18^b^
C18:2 ω6	49.68±1.03	18.36±0.4#	19.36±0.62	18.28±0.39
C18:3 ω3	5.53±0.75	1.29±0.09# a	15.21±0.66^b^	1.31±0.1^a^
C20:4 ω6	0.29±0.06	0.22±0.05	0.17±0	0.17±0.01
C22:0	-	0.05±0.01	0.12±0.02	0.13±0.01
C20:5 ω3	-	0.01±0	0.01±0	0.01±0
C22:5 ω3	-	0.06±0.01	0.04±0	0.03±0
C22:6 ω3	-	0.02±0	0.01±0	0.012±0
Σ PUFA	55.5±2.13	19.96±0.98# a	34.8±1.2^b^	19.81±0.9^a^
Σ ω6	49.97±3.4	18.58±2.7#	19.53±2.1	18.45±1.87
Σ ω3	5.53±0.12	1.38±0.09# a	15.27±1.18^b^	1.36±0.08^a^
ω6:ω3 ratio	9.03:1	13.46:1	1.27:1	13.56:1

CT, control diet; FS 10%, flax seed oil substituted 10%; HF, high fat diet; MUFA, monounsaturated fatty acid; OL 10%, olive oil substituted 10%; PUFA, polyunsaturated fatty acid; SFA, saturated fatty acid.

# , mean significant difference between CT an HF groups by Student’s test (*P<0.05*).

a–c , mean values followed by the same letter in the line are not different by Tukey’s test (*P< 0.05*).

Σ SFA, includes 8:0, 10:0, 12:0 and 17:0.

Σ MUFA, includes 14:1, 17:1, 20:1(ω-7) and 24:1.

Σ PUFA, includes 18:3(ω-6), 20:2(ω-6), 20:3(ω-6) and 22:5(ω-6).

### Impact of diet change on blood and hypothalamic fatty acids

Consumption of the HF diet led to significant increases of C18:0, C20:0 and C22:0, and to a significant reduction of total ω3 in the blood, as compared to mice fed on chow (Table 3). The 10% FS substitution resulted in a significant increase of total ω3 content in the blood and a reduction of the ω6∶ω3 ratio from 7.1 to 3.2, as compared to mice fed on HF (Table 3). The 10% OL substitution resulted in no significant increase of total ω9 content in the blood, but produced a significant reduction of total blood content of saturated fatty acids, as compared to mice fed on HF (Table 3). In the hypothalamus, the HF diet produced significant increases of C20:0 and C22:0, as compared to mice fed on chow (Table 4). The 10% FS substitution resulted in reductions of the C20:0 and C22:0 levels and reduction of the ω6∶ω3 ratio from 1∶1.29 to 1∶1.65 in the hypothalamus, as compared to mice fed on HF (Table 4). The 10% OL substitution resulted in significant reductions of the relative C20:0 and C22:0 contents in the hypothalamus, as compared to mice fed on HF (Table 4).

**Table 3:** Fatty acid composition of the blood (% of total fat).

Fatty Acids	CT	HF	FS 10%	OL 10%
C14:0	0.092±0.009	0.167±0.06	0.165±0.06	0.152±0.02
C16:0	19.4±0.82	16.7±0.2	16.42±1.03	17.01±0.91
C18:0	10.45±0.57	12.04±0.45#	11.53±0.85	11.45±0.85
C20:0	0.02±0.002	0.135±0.12#	0.17±0.04	0.182±0.04
C22:0	0.03±0.004	0.132±0.09#	0.167±0.03	0.097±0.02
Σ SFA	29.92±0.35	29.18±0.2	28.78±1.5	28.97±0.79
C16:1 ω7	0.742±0.09	0.855±0.1	1.085±0.2	1.035±0.13
C18:1 ω9	11.62±0.59	12.42±3.5	13.31±1.12	15.17±1.53
Σ MUFA	12.10±0.65	13.97±3.7	14.4±1.32	16.25±1.51
C18:2 ω6	31.77±0.87	21.92±1.2#	25.47±3.19	19.1±0.66
C18:3 ω3	0.567±0.1	0.142±0.03# a	1.825±0.85^b^	0.187±0.05^a^
C20:4 ω6	18.93±0.63	26,19±5.05# a	15.22±2.68^b^	26.82±1.79^a^
C20:5 ω3	0.567±0.05	0.3±0.06	0.344±0.94	0.242±0.02
C22:5 ω3	0.377±0.18	0.357±0.11^a^	0.82±0.21^b^	0.305±0.11^c^
C22:6 ω3	5.9±0.18	6.97±1.1	6.705±0.24	5.36±0.61
Σ PUFA	57.62±0.36	54.68±4.02	54.0±2.06	52.02±1.58
Σ ω6	50.20±0.56	47.78±3.95^a^	41.27±0.85^b^	45.9±1.3^a^ ^b^
Σ ω3	7.412±0.34	6.897±1.1^a^	12.7±1.7^b^	6.097±0.63^a^
ω6:ω3 ratio	6.95:1	7.1:1	3.2:1	7.7:1

CT, control diet; FS 10%, flax seed oil substituted 10%; HF, high fat diet; MUFA, monounsaturated fatty acid; OL 10%, olive oil substituted 10%; PUFA, polyunsaturated fatty acid; SFA, saturated fatty acid.

# , mean significant difference between CT an HF groups by Student’s test (*P<0.05*).

a–b , mean values followed by the same letter in the line are not different by Tukey’s test (*P< 0.05*).

Σ SFA, includes 8:0, 10:0, 12:0 and 17:0.

Σ MUFA, includes 14:1, 17:1, 20:1(ω-7) and 24:1.

Σ PUFA, includes 18:3(ω-6), 20:2(ω-6), 20:3(ω-6) and 22:5(ω-6).

**Table 4:** Fatty acid composition of the hypothalamus (% of total fat).

Fatty Acids	CT	HF	FS 10%	OL 10%
C14:0	0.15±0.03	0.177±0.05	0.11±0.05	0.14±0.03
C16:0	22.85±1.28	22.28±0.68	24.78±1.27	23.47±0.98
C18:0	21.4±0.51	20.63±0.19	22±1.1	21.78±1.1
C20:0	0.355±0.09	0.42±0.07# a	0.244±0.12^b^	0.22±0.015^a^
C22:0	0.23±0.04	0.24±0.01# a	0.155±0.01^b^	0.13±0.08^b^
Σ SFA	44.9±1.9	44.33±1.1	45.6±0.26	44.56±1.8
C16:1 ω7	0.805±0.03	0.75±0.02	0.877±0.19	0.8±0.2
C18:1 ω9	24.98±1.65	25.17±0.54	24.05±3.16	23.07±2.12
Σ MUFA	26.75±0.18	30.02±0.46# a	26.72±3.76^a^	25.76±0.95^b^
C18:2 ω6	1.9±0.71	2.56±0.1	-	-
C18:3 ω3	0.07±0.02	0.12±0.002	-	-
C20:4 ω6	10.17±0.78	9.73±0.61	10.08±2.87	11.53±2.23
C20:5 ω3	0.22±0.04	0.3±0.07^a^	0.17±0.07^b^	0.177±0.07^b^
C22:5 ω3	0.31±0.09	0.14±0.04# a	0.28±0.11^b^	0.16±0.09^a^
C22:6 ω3	15.67±0.72	15.33±0.24	16.27±1.02	17.7±1.4
Σ PUFA	28.36±0.92	25.6±0.54# a	27.67±4.03^a^ ^b^	29.62±0.98^b^
Σ ω6	12.1±0.11	12.3±2.16	10.13±2	11.57±1.9
Σ ω3	16.3±0.7	15.9±1.11	16.74±1.14	18.04±1.15
ω6:ω3 ratio	1.0:1.34	1.0:1.29	1.0:1.65	1.0:1.55

CT, control diet; FS 10%, flax seed oil substituted 10%; HF, high fat diet; MUFA, monounsaturated fatty acid; OL 10%, olive oil substituted 10%; PUFA, polyunsaturated fatty acid; SFA, saturated fatty acid.

# , mean significant difference between CT an HF groups by Student’s test (*P<0.05*).

a–b , mean values followed by the same letter in the line are not different by Tukey’s test (*P< 0.05*).

Σ SFA, includes 8:0, 10:0, 12:0 and 17:0.

Σ MUFA, includes 14:1, 17:1, 20:1(ω-7) and 24:1.

Σ PUFA, includes 18:3(ω-6), 20:2(ω-6), 20:3(ω-6) and 22:5(ω-6).

### FS and OL substitutions in the diet reduce food intake and body mass gain

**Figure 2:**
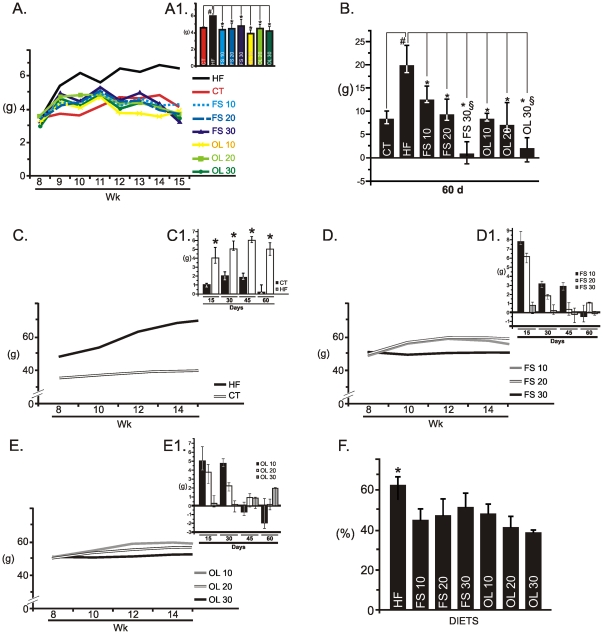
Food intake and body mass variation. A, Mean daily spontaneous food intake (g) of Swiss mice fed on regular chow (CT), high-fat diet (HF), flax seed- (FS) or olive oil- (OL) substituted (10, 20 or 30%) diets for eight weeks; results are depicted as daily food intake along the time (A), and as the means obtained during the whole period (A1). B, Body mass variation for each group during the whole experimental period. C-E, Body mass variation (g) during the 60-day experimental period (C-E) or during each of the four 15-day experimental periods (C1-E1) for CT and HF groups (C, C1); for the FS substituted groups (D, D1); and for the OL substituted groups (E, E1). F, Diet preference assay, lean Swiss mice were fasted for 10 h and then similar amounts of CT or HF (HF) diets were offered; the same approach was used to compare the preference for each of the FS or OL substituted diets against HF; results are presented as the relative caloric consumption of the tested diet during 12h. In all experiments, n = 5; in A and B, #p<0.05 *vs.* CT and *p<0.05 *vs.* HF; in C and F *p<0.05 *vs.* CT; in B, §p<0.05 *vs.* FS10 or OL10.

In order to evaluate the impact of the dietary fatty acid composition on a number of metabolic parameters, mice were initially fed on a saturated-rich HF diet for 8 weeks and then randomly assigned to diets containing stepwise substitution fractions of FS and OL, according to Table 1. The substitution of diet lard by FS or OL significantly reduced food intake, independently of oil type and diet composition (Fig. 2A). The impact of diet fatty acid substitution on body mass variation was dependent on composition but not on unsaturated fatty acid type. Thus, increasing the fraction of unsaturated fatty acid in the diet significantly reduced the body mass gain during the 60-day experimental period (Fig. 2B). Interestingly, when evaluating body mass variation during the four 15-day periods that spanned the whole experimental time, it was evident that diets containing lower fractions of unsaturated fatty acids (10 and 20%) led to an initial body mass increase, which was followed by reduction in body mass gain, or even loss of body mass. This was not observed in diets containing 30% FS or OL, which led to low body mass gain or even body mass loss, in all the four 15-day periods (Fig. 2C–2E). The effects herein described were not due to palatability, because except for a discrete preference for HF against chow, no differences in preference were found when comparing each of the unsaturated fatty acid substitutions against HF (Fig. 2F).

### FS and OL substitutions in the diet improve insulin action and glucose homeostasis

**Figure 3:**
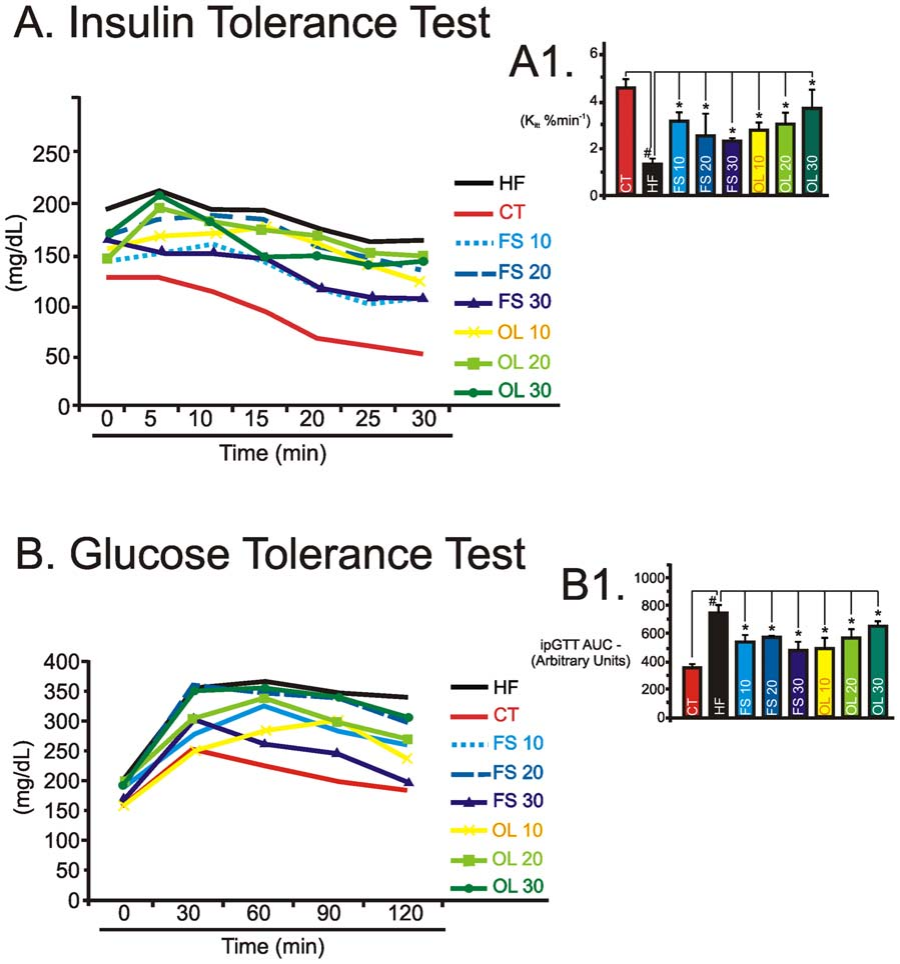
Metabolic parameters. Blood glucose levels (A), and constant for glucose decay during an insulin tolerance test (Kitt) (%/min) (A1); and, blood glucose levels (B) and the area under glucose curve (AUC) (B1) during an intraperitoneal glucose tolerance test (ipGTT) were obtained at the end of an eight-week experimental period for Swiss mice fed on regular chow (CT), high-fat diet (HF), flax seed- (FS) or olive oil- (OL) substituted (10, 20 or 30%) diets. In all experiments, n = 5; #p<0.05 *vs.* CT and *p<0.05 *vs.* HF.

In humans and experimental animals, the consumption of high-fat diets leads to insulin resistance and glucose intolerance [23], [24]. To determine the impact of unsaturated fatty acid substitution on insulin action and glucose homeostasis, mice fed on the distinct unsaturated fatty acid-substituted diets were submitted to insulin and glucose tolerance tests. As depicted in Figures 3A and 3B, unsaturated fatty acid substitution, irrespectively of substitution fraction or fatty acid type, produced significant reductions in K*itt* and the glucose area under curve, during a GTT, respectively.

### FS and OL substitutions reduce diet-induced hypothalamic inflammation and correct the response to nutrient sensing signals

**Figure 4:**
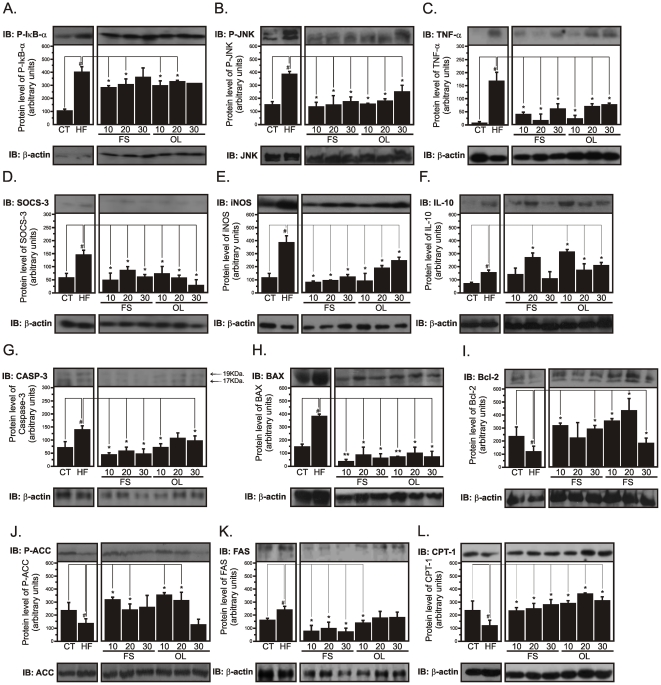
Signal transduction in the hypothalamus. Hypothalamic total protein extracts obtained from Swiss mice fed on regular chow (CT), high-fat diet (HF), flax seed- (FS) or olive oil- (OL) substituted (10, 20 or 30%) diets for eight weeks were used in immunoblotting (IB) experiments to evaluate protein expression and/or activity. Specific antibodies against phospho-IκB-α (P-IκBα) (A), phospho-JNK (P-JNK) (B), TNF-α (C), SOCS-3 (D), iNOS (E), IL-10 (F), Caspase-3 (CASP-3) (G), BAX (H), Bcl-2 (I), phospho-ACC (P-ACC) (J), FAS (K) and CPT-1 (L) were used to identify respective protein targets. Loading was evaluated by re-probing the membranes with anti-β-actin (A, C-I, K and L), anti-JNK (B) or anti-ACC (J) antibodies. In all experiments, n = 5; #p<0.05 *vs.* CT and *p<0.05 *vs.* HF.

A number of previous studies have shown the effect of saturated-rich high-fat diets on the induction of hypothalamic inflammation [4], [5], [8]. The induction of inflammatory gene expression in the hypothalamus can be detected as early as 2w after the introduction of the HF diet (Figure S1). The substitution of saturated by unsaturated fatty acids in the diet promoted dose-, and fatty acid type-independent reductions in the expressions of the inflammatory markers pIκB-α (Fig. 4A), pJNK (Fig. 4B), TNF-α (Fig. 4C), SOCS3 (Fig. 4D) and iNOS (Fig. 4E), while increasing the expression of the anti-inflammatory cytokine, IL-10 (Fig. 4F), in the hypothalami of mice. In addition, ω3- and ω9-rich diets led to up to 3-fold increases in the hypothalamic expression of the anti-apoptotic protein Bcl-2 (Fig 4I), while reducing the apoptotic proteins, caspase-3 (Fig. 4G) and Bax (Fig. 4H). All these effects were accompanied by the inhibition of ACC activity (Fig. 4J), while decreasing the expression of FAS (Fig. 4K) and increasing CPT-1 (Fig. 4L).

### Icv ω3 and ω9 treatments reduce food intake and body adiposity

**Figure 5:**
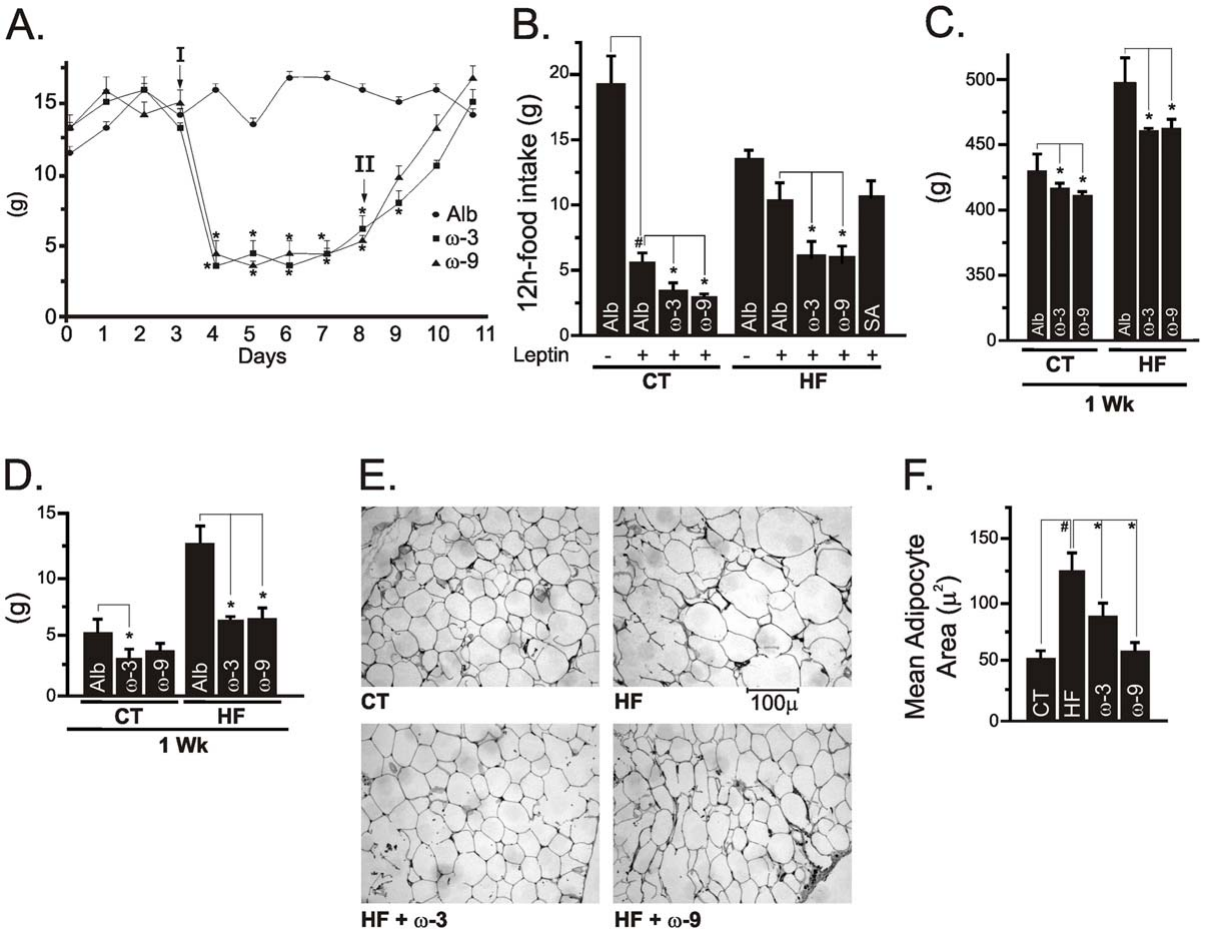
Food intake, body mass and adiposity in icv-treated rats. Wistar rats fed on a regular chow (CT) or on a high-fat diet (HF) were icv cannulated and treated for five (A) or seven (B-F) days with diluent (albumin, Alb), ω3-, ω9-fatty acids or stearic acid (SA) and then used for determination of feeding behavior and adiposity. A, daily food intake (g) of rats treated icv with Alb (filled circles), ω3 (filled squares) or ω9 (filled triangles) fatty acids for five days; the beginning (I) and the end (II) of treatment are labeled with arrows. B, The suppression of spontaneous food intake (g) by leptin was evaluated at the end of the experimental period. C, Body mass variation (g) during the seven-day icv treatment period. D, Epididymal fat mass (g) at the end of the experimental period. E, Histological evaluation (hematoxilin-eosin staining of 5 µm sections) of epididymal fat. F, Mean adipocyte area obtained from histological sections. In all experiments, n = 5. In A, C and D, *p<0.05 *vs.* Alb; in B, #p<0.05 *vs.* Alb(−) and *p<0.05 *vs.* Alb(+); in F, #p<0.05 *vs.* CT, *<0.05 *vs.* HF.

To determine the direct effect of unsaturated fatty acids on hypothalamic function, rats were submitted to icv cannulation and treated with ω3, ω9 or stearic acid for seven days. Daily icv injections of ω3 or ω9 fatty acids significantly reduced spontaneous food intake (Fig. 5A) and enhanced the anorexigenic effect of leptin in both control and HF rats (Fig. 5B). This resulted in significant body mass loss (Fig. 5C) and reduction of epididymal fat content (Fig. 5D), with reduction of mean adipocyte diameter (Fig. 5E and 5F). Discontinuation of the icv injections with either unsaturated fatty acid resulted in a complete restoration of base-line feeding behavior (Fig. 5A).

### Icv ω3 and ω9 treatments reduce hypothalamic inflammation and the expression of apoptotic proteins

**Figure 6:**
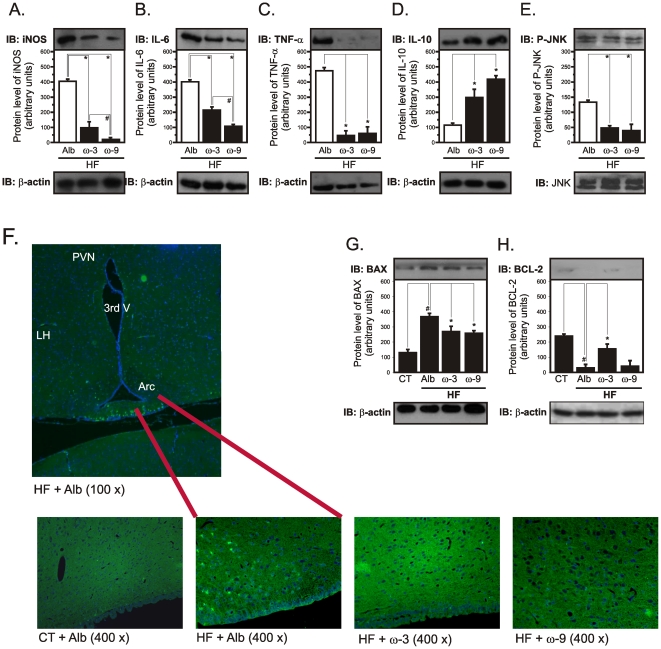
Expression of inflammatory and apoptotic proteins in the hypothalamus of icv-treated rats. Wistar rats fed on a regular chow (CT) or on a high-fat diet (HF) and icv cannulated were treated for seven days with diluent (albumin, Alb), ω3 or ω9 fatty acids and then used in immunoblotting (IB) and immufluorescence experiments. Specific antibodies against iNOS (A), IL-6 (B), TNF-α (C), IL-10 (D), phospho-JNK (P-JNK) (E), BAX (G), and Bcl-2 (H) were used to identify respective protein targets in hypothalamic samples. Loading was evaluated by re-probing the membranes with anti-β-actin (A-D, G and H) or anti-JNK (E). In F, 5 µm sections of the hypothalamus were labeled with an anti-F4/80 antibody. In all experiments, n = 5. In A-E, *p<0.05 *vs.* Alb; in A and B, #p<0.05 *vs.* ω3; In G and H, #p<0.05 *vs.* CT, *<0.05 *vs.* Alb.

Seven days of ω3 or ω9 icv treatment significantly reduced the hypothalamic expression of markers of inflammation, iNOS (Fig. 6A), IL-6 (Fig. 6B), TNF-α (Fig. 6C) and pJNK (Fig. 6E). The ω9 fatty acid was significantly more effective in reducing iNOS (Fig. 6A) and IL-6 (Fig. 6B), while ω3 fatty acid was significantly more effective in reducing pJNK (Fig. 6E). TNF-α was similarly affected by both unsaturated fatty acids (Fig. 6C). The reduction in hypothalamic inflammation, induced by ω3 and ω9 fatty acids, was also detected as a reduction in F4/80-positive cells in the arcuate nucleus (Fig. 6F). In addition, the hypothalamic expression of the anti-inflammatory cytokine, IL-10 was significantly increased by either ω3 or ω9 fatty acids (Fig. 6D). Moreover, both icv ω3 and ω9 produced a significant reduction in the hypothalamic expression of the pro-apoptotic protein, Bax (Fig. 6G), while ω3, but not ω9 fatty acid produced an increase in the expression of the anti-apoptotic protein, Bcl-2 (Fig. 6H).

### Icv ω3 and ω9 treatment improve anorexigenic and pro-thermogenic signaling in the hypothalamus

**Figure 7:**
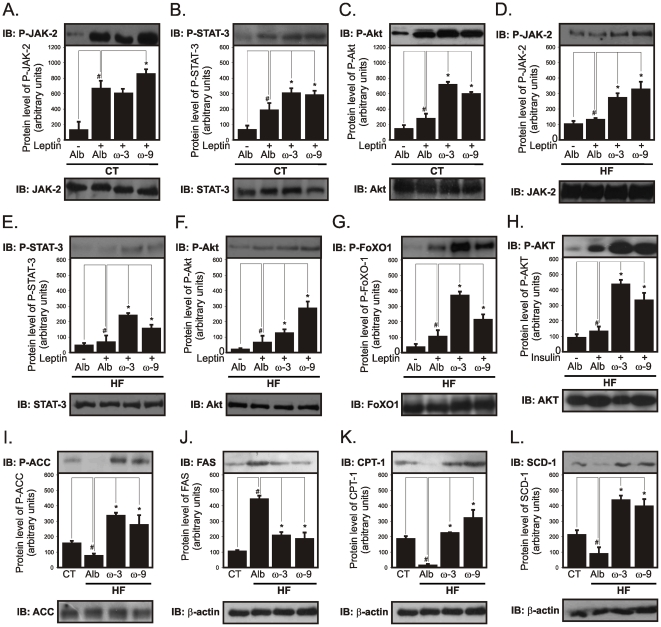
Effect of icv ω3 and ω9 on hypothalamic signaling. Wistar rats fed on a regular chow (CT) or on a high-fat diet (HF) and icv cannulated were treated for seven days with diluent (albumin, Alb), ω3 or ω9 fatty acids. In addition, in some experiments, rats were acutely treated with a single dose of either leptin (2 µl, 10^−6^M: A-G) or insulin (2 µl, 10^−6^M: H) and then used in immunoblotting (IB) experiments. Specific antibodies against phospho-JAK2 (P-JAK2) (A and D), phospho-STAT3 (P-STAT3) (B and E), phospho-Akt (P-Akt) (C, F and H), phospho-FoxO1 (P-FoxO1) (G), phospho-ACC (P-ACC) (I), FAS (J), CPT-1 (K) and SCD-1 (L) were used to identify respective protein targets in hypothalamic tissue. Loading was evaluated by re-probing the membranes with anti-β-actin (J-L), anti-JAK2 (A and D), anti-STAT3 (B and E), anti-Akt (C, F and H), anti-FoxO1 (G) or anti-ACC (I). In A-H, #p<0.05 *vs.* Alb (−), *p<0.05 *vs.* Alb (+); in I-L, #p<0.05 *vs.* CT, *p<0.05 *vs.* Alb.

**Figure 8:**
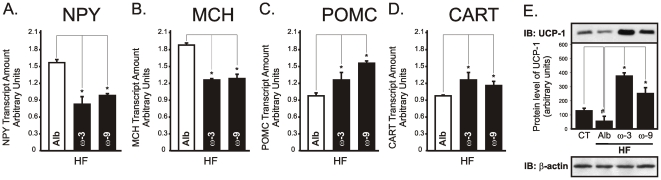
Effect of icv ω3 and ω9 on neurotransmitter expression and thermogenesis. Wistar rats fed on a regular chow (CT) or on a high-fat diet (HF) and icv cannulated were treated for seven days with diluent (albumin, Alb), ω3 or ω9 fatty acids and then used in real-time PCR and immunobloting experiments. Total RNA obtained from hypothalami were used in real-time PCR to amplify the mRNAs of NPY (A), MCH (B), POMC (C), and CART (D). Brown adipose tissue total protein extracts were used for evaluation of UCP-1 expression by immunoblot (E). In all experiments, n = 5. In A-D, *p<0.05 *vs.* Alb (−); in E, #p<0.05 *vs.* CT, *p<0.05 *vs.* Alb.

Leptin and insulin provide the most robust anorexigenic and pro-thermogenic signals to the hypothalamus [25], [26]. The icv treatment of rats with either ω3 or ω9 fatty acids significantly improved leptin signal transduction through JAK2 (Fig. 7A), STAT3 (Fig. 7B) and Akt (Fig. 7C) in lean rats. In addition, in HF rats, leptin signaling through JAK2 (Fig. 7D), STAT3 (Fig. 7E), Akt (Fig. 7F) and FOXO1 (Fig. 7G), as well as insulin signal transduction through Akt (Fig. 7H), were significantly improved. Proteins belonging to the hypothalamic nutrient sensing pathway were also modulated. Thus, ω3 and ω9 fatty acids produced an increase in ACC phosphorylation (Fig. 7I) accompanied by a reduction in FAS expression (Fig. 7J) and increased CPT1 (Fig. 7K) and SCD1 (Fig. 7L) expressions. All these effects were accompanied by reductions in the mRNA expressions of NPY (Fig. 8A) and MCH (Fig. 8B) and by the increase of POMC (Fig. 8C) and CART (Fig. 8D). The pro-thermogenic effect of the treatment with ω3 and ω9 was further evidenced by the increased expression of UCP1 in BAT (Fig. 8E).

### Unsaturated fatty acids activate signal transduction through GPR120 in the hypothalamus

**Figure 9:**
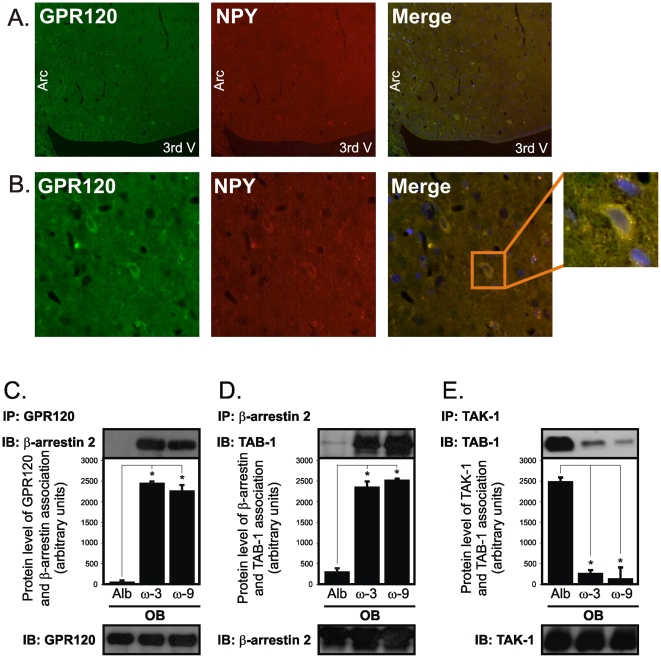
GPR120 signal transduction in the hypothalamus. Five µm sections of the hypothalamus obtained from obese Wistar rats were labeled with anti-GPR120 (green) and NPY (red) antibodies, low (A) and high (B) magnifications are depicted. Icv cannulated obese Wistar rats were acutely treated with diluent (albumin, Alb), ω3 or ω9 fatty acids and then used in immunoprecipitation (IP)/immunoblotting (IB) experiments employing antibodies against GRP120 (C), β-arrestin 2 (C and D), TAK1 (E), and TAB1 (D and E). In all experiments n = 5. In C-E, *p<0.05 *vs.* Alb.

A recent study has identified GPR120 as the receptor mediating the anti-inflammatory and insulin-sensitizing actions of ω3 fatty-acids in monocytes [27]. To investigate if ω3 and ω9 fatty acids could act through the same receptor in the hypothalamus we first performed double-labeling immunofluorescence studies, which revealed the co-expression of GPR120 and NPY in cells of the arcuate nucleus (Fig. 9A and 9B). Next, we acutely injected ω3 or ω9 fatty acids icv and evaluated signal transduction through GPR120. Upon either ω3 or ω9 stimulation, GPR120 undergoes a rapid association with the scaffolding protein β-arrestin 2 (Fig. 9C), which is immediately followed by the association of β-arrestin 2 with TAB1 (Fig. 9D). Simultaneously, TAB1 dissociates from TAK1 (Fig. 9E), forwarding the GPR120 signal downstream.

## Discussion

The present study was undertaken to evaluate the effect of the nutritional substitution of saturated by unsaturated fatty acids, and also the direct effect of unsaturated fatty acids on the modulation of hypothalamic inflammation and function in obesity. In modern societies, the progressive increase in the dietary consumption of saturated fatty acids is taken as one of the main determinants of obesity, and dietetic approaches aimed at substituting saturated by unsaturated fats have succeeded in reducing body mass and improving a number of metabolic parameters in overweight patients [23].

Saturated fatty acids, acting directly in the hypothalamus or as a component of the diet, induce inflammation by activating TLR4 signaling and ER stress, which leads to increased inflammatory gene transcription, hypothalamic dysfunction and eventually neuronal apoptosis [5], [6]. These local effects are accompanied by progressive loss of the balance between food intake and energy expenditure, which result in increased adiposity and metabolic breakdown [2], [28].

Due to the known anti-inflammatory actions of unsaturated fatty acids in other tissues or cell-types [12], [13], [14], we hypothesized that, by acting as nutrients and/or directly in the hypothalamus, these molecules could, at least partially, reduce inflammation and correct hypothalamic activity in the regulation of energy homeostasis. In the first part of the study, diet-induced obese mice were fed diets composed of a stepwise substitution of the saturated by unsaturated fatty acids. The substitutions of the fatty acid components in the diet resulted in increased relative amounts of unsaturated fatty acids, predominating oleic acid in the olive oil substituted diets and linolenic acid in the flax seed oil substituted diets. The FS substitution resulted in increased relative amount of ω3 in the blood and hypothalamus. However, OL substitution did not impact on the relative ω9 amount in either tissue. Instead, a reduction of saturated fat was achieved. We have no current explanation for that, but we suspect that the overall metabolism of ω9 avoids its excessive increase in the hypothalamic tissue. This is supported by the fact that the diets modulate enzymes involved in fatty acid metabolism such as FAS and SCD1, as shown by our experiments. As a consequence of fatty acid substitution there was a reduction in food intake to levels similar to control, an effect that was independent of fatty acid type and quantity. However, this was accompanied by a dose-dependent-like effect on body mass, with maximum effect in the 30% substituted diets, irrespectively of fatty acid type. Interestingly, most of the effect of the substituted diets occurred during the second half of the experimental period. In fact, in some conditions, mice presented an increase in body mass gain during the first 30 days. Nevertheless, even the groups presenting body mass gain at the beginning, reached the end of the experimental period with a significantly lower body mass gain, as compared to controls.

We next evaluated the effect of fatty acid substitution on whole body insulin action and glucose homeostasis. Similarly to the effect of the substituted diets on food intake, here, a complete restoration of insulin action and glucose homeostasis to levels similar to those of lean controls was obtained, independently of diet composition and fatty acid type. Since the effect of substitutions on body mass were dependent on the quantity of the unsaturated fatty acids, we believe the effect on insulin action and glucose homeostasis was simply due to the presence of the increased amounts of unsaturated fatty acids in the diets and not secondary to body mass reduction. In this regard, studies by L. Rossetti's group have shown the direct effect of central administration of oleic acid on the control of peripheral glucose homeostasis, which depends, at least in part, on the neural control of hepatic gluconeogenesis [29], [30], [31].

Recent data have increased interest in the effects of hypothalamic inflammation on whole body energy homeostasis [28]. The activation of inflammatory signaling in this particular anatomical region is capable of modulating glucose homeostasis and thermogenesis, mostly by controlling neural inputs to specific organs [31], [32]. This mechanism, acts in concert with inflammation-induced hypothalamic resistance to the anorexigenic hormones, leptin and insulin, to produce the multiple components of the complex phenotype observed in obesity. The reduction of hypothalamic inflammation in obesity corrects simultaneously feeding, thermogenesis and metabolic disarrangements, placing this phenomenon in a central position in the pathogenesis of obesity [4], [5], [8]. Here, when mice were fed unsaturated fatty acid-rich diets, a reduction in the hypothalamic expression of a number of inflammatory markers was achieved, which is in accordance with the results obtained in animal models treated with anti-inflammatory drugs [4], [5], [11], or harboring genetic modifications that impair inflammatory signaling [5], [8]. In addition, the expression of pro-apoptotic proteins was decreased, while the signaling through the AMPK/ACC pathway was corrected, showing that the effect of the unsaturated fatty acid was broad, restoring, at least in part, all defects reported so far in animal models of obesity [4], [5], [6], [33].

To determine whether the anti-inflammatory and metabolic effects observed with the diets were indeed due to the presence of increased amounts of unsaturated fatty acids, we next evaluated the direct effect of ω3 and ω9 in the hypothalamus. Both fatty acids induced a large reduction in spontaneous food intake, which was completely restored after discontinuation. A similar effect has been previously reported for oleic acid [31], and now we have extended this observation to linolenic acid. As a consequence of the reduction in feeding, both fatty acids promoted reductions in adiposity, with a reduction in mean adipocyte size. Of great importance, the fatty acids exerted a potent anti-inflammatory effect, reducing the hypothalamic expression of cytokines and inflammatory signaling proteins. In addition, the presence of activated microglial cells was restrained by either ω3 or ω9 icv treatments.

The effect of icv unsaturated fatty acids was not restricted to the inflammatory pathways, and significant improvement of leptin and insulin signal transduction in the hypothalamus was also achieved. This is of major interest in obesity because the impairment of signal transduction through these pathways in the hypothalamus seems to constitute the molecular basis for obesity [2], [34], [35], [36]. In addition, as for the diets, icv unsaturated fatty acids corrected signal transduction through the AMPK/ACC pathway. This pathway is involved in nutrient sensing and cross-talks with hormone signaling systems to control neurotransmitter expression [33], [37].

Next, we determined the impact of the unsaturated fatty acids on the hypothalamic expression of neurotransmitters involved in the control of energy homeostasis. In general, animal models of obesity present increased expressions of the orexigenic, anti-thermogenic NPY and MCH and reduced expressions of the anorexigenic, pro-thermogenic POMC and CART [38], [39]. Either ω3 or ω9 fatty acids reduced hypothalamic expression of NPY and MCH while increasing the expressions of POMC and CART. These results are somewhat similar to those achieved by other anti-inflammatory approaches [5], [8]. Interestingly, both fatty acids significantly increased the expression of UCP1 in the BAT, suggesting that an increase in thermogenesis may play a role in the phenotypic changes obtained. In other animal models of obesity, anti-inflammatory approaches also produced beneficial changes in the expression of UCP1 in BAT [40].

Finally, we showed that the unsaturated fatty acid receptor, GPR120 is expressed in the hypothalamus and is activated in response to ω3 and ω9 fatty acids. GPR120 was recently identified as an important mediator of the anti-inflammatory and insulin-sensitizing effects of unsaturated fatty acids in monocytes [27]. Upon ligand binding, GPR120 activates signal transduction through β-arrestin 2/TAB1, which switches-off the TLR4 and TNF inflammatory pathways, these effects were reproduced in the hypothalamus of obese rats. In addition, we show that GPR120 is expressed in NPY expressing neurons. We decided to test this particular co-expression because of the remarkable effect of the unsaturated fatty acids to reduce NPY expression, however, cells other than the NPY neurons were also stained by the GPR120 antibody (not shown). Since NPY is inhibited in response to leptin, and, in diet-induced obesity there is resistance to leptin action in the hypothalamus, it possible that upon unsaturated fatty acid stimulation, GPR120 present in NPY neurons can play a role reestablishing the action of leptin towards NPY gene expression inhibition.

In conclusion, unsaturated fatty acids can reproduce a number of the anti-inflammatory effects of TLR4 or TNF-α inhibition and, therefore, constitute an attractive nutritional approach to treat obesity. At least part of this effect may be mediated by the GPR120 receptor.

## Supporting Information

Figure S1 Time course of high-fat diet (HF)-induced expression of inflammatory markers in the hypothalamus. Swiss mice fed on chow (CT) or HF for 2, 4 or 8 weeks were employed for determination of the hypothalamic expression of interleukin-1b (A-C) or tumor necrosis factor-a (D-E) transcripts by real-time PCR. In all experiments, n = 5; *p<0.05 *vs.* CT.
(TIF)

## References

[1] Yang L, Hotamisligil GS (2008). Stressing the brain, fattening the body.. Cell.

[2] Velloso LA, Araujo EP, de Souza CT (2008). Diet-induced inflammation of the hypothalamus in obesity.. Neuroimmunomodulation.

[3] Wisse BE, Schwartz MW (2009). Does hypothalamic inflammation cause obesity?. Cell Metab.

[4] De Souza CT, Araujo EP, Bordin S, Ashimine R, Zollner RL (2005). Consumption of a fat-rich diet activates a proinflammatory response and induces insulin resistance in the hypothalamus.. Endocrinology.

[5] Milanski M, Degasperi G, Coope A, Morari J, Denis R (2009). Saturated fatty acids produce an inflammatory response predominantly through the activation of TLR4 signaling in hypothalamus: implications for the pathogenesis of obesity.. J Neurosci.

[6] Moraes JC, Coope A, Morari J, Cintra DE, Roman EA (2009). High-fat diet induces apoptosis of hypothalamic neurons.. PLoS One.

[7] Zabolotny JM, Kim YB, Welsh LA, Kershaw EE, Neel BG (2008). Protein-tyrosine phosphatase 1B expression is induced by inflammation in vivo.. J Biol Chem.

[8] Zhang X, Zhang G, Zhang H, Karin M, Bai H (2008). Hypothalamic IKKbeta/NF-kappaB and ER stress link overnutrition to energy imbalance and obesity.. Cell.

[9] Bjorbak C, Lavery HJ, Bates SH, Olson RK, Davis SM (2000). SOCS3 mediates feedback inhibition of the leptin receptor via Tyr985.. J Biol Chem.

[10] Howard JK, Cave BJ, Oksanen LJ, Tzameli I, Bjorbaek C (2004). Enhanced leptin sensitivity and attenuation of diet-induced obesity in mice with haploinsufficiency of Socs3.. Nat Med.

[11] Araujo EP, De Souza CT, Ueno M, Cintra DE, Bertolo MB (2007). Infliximab restores glucose homeostasis in an animal model of diet-induced obesity and diabetes.. Endocrinology.

[12] Lee TH, Hoover RL, Williams JD, Sperling RI, Ravalese J, 3rd (1985). Effect of dietary enrichment with eicosapentaenoic and docosahexaenoic acids on in vitro neutrophil and monocyte leukotriene generation and neutrophil function.. N Engl J Med.

[13] Endres S, Ghorbani R, Kelley VE, Georgilis K, Lonnemann G (1989). The effect of dietary supplementation with n-3 polyunsaturated fatty acids on the synthesis of interleukin-1 and tumor necrosis factor by mononuclear cells.. N Engl J Med.

[14] Reynolds CM, Draper E, Keogh B, Rahman A, Moloney AP (2009). A conjugated linoleic acid-enriched beef diet attenuates lipopolysaccharide-induced inflammation in mice in part through PPARgamma-mediated suppression of toll-like receptor 4.. J Nutr.

[15] Bonora E, Moghetti P, Zancanaro C, Cigolini M, Querena M (1989). Estimates of in vivo insulin action in man: comparison of insulin tolerance tests with euglycemic and hyperglycemic glucose clamp studies.. J Clin Endocrinol Metab.

[16] Johnson AK, Epstein AN (1975). The cerebral ventricles as the avenue for the dipsogenic action of intracranial angiotensin.. Brain Res.

[17] Weinstein JR, Swarts S, Bishop C, Hanisch UK, Moller T (2008). Lipopolysaccharide is a frequent and significant contaminant in microglia-activating factors.. Glia.

[18] Martins EF, Miyasaka CK, Newsholme P, Curi R, Carpinelli AR (2004). Changes of fatty acid composition in incubated rat pancreatic islets.. Diabetes Metab.

[19] Bertelli DF, Araujo EP, Cesquini M, Stoppa GR, Gasparotto-Contessotto M (2006). Phosphoinositide-specific inositol polyphosphate 5-phosphatase IV inhibits inositide trisphosphate accumulation in hypothalamus and regulates food intake and body weight.. Endocrinology.

[20] Paxinos G, Watson CR, Emson PC (1980). AChE-stained horizontal sections of the rat brain in stereotaxic coordinates.. J Neurosci Methods.

[21] Kim MS, Pak YK, Jang PG, Namkoong C, Choi YS (2006). Role of hypothalamic Foxo1 in the regulation of food intake and energy homeostasis.. Nat Neurosci.

[22] Bradford MM (1976). A rapid and sensitive method for the quantitation of microgram quantities of protein utilizing the principle of protein-dye binding.. Anal Biochem.

[23] Rivellese AA, Lilli S (2003). Quality of dietary fatty acids, insulin sensitivity and type 2 diabetes.. Biomed Pharmacother.

[24] De Souza CT, Araujo EP, Stoppiglia LF, Pauli JR, Ropelle E (2007). Inhibition of UCP2 expression reverses diet-induced diabetes mellitus by effects on both insulin secretion and action.. Faseb J.

[25] Plum L, Belgardt BF, Bruning JC (2006). Central insulin action in energy and glucose homeostasis.. J Clin Invest.

[26] Myers MG (2004). Leptin receptor signaling and the regulation of mammalian physiology.. Recent Prog Horm Res.

[27] Oh DY, Talukdar S, Bae EJ, Imamura T, Morinaga H (2010). GPR120 is an omega-3 fatty acid receptor mediating potent anti-inflammatory and insulin-sensitizing effects.. Cell.

[28] Thaler JP, Choi SJ, Schwartz MW, Wisse BE Hypothalamic inflammation and energy homeostasis: resolving the paradox.. Front Neuroendocrinol.

[29] Lam TK, Gutierrez-Juarez R, Pocai A, Bhanot S, Tso P (2007). Brain glucose metabolism controls the hepatic secretion of triglyceride-rich lipoproteins.. Nat Med.

[30] Morgan K, Obici S, Rossetti L (2004). Hypothalamic responses to long-chain fatty acids are nutritionally regulated.. J Biol Chem.

[31] Obici S, Feng Z, Morgan K, Stein D, Karkanias G (2002). Central administration of oleic acid inhibits glucose production and food intake.. Diabetes.

[32] Arruda AP, Milanski M, Romanatto T, Solon C, Coope A Hypothalamic actions of tumor necrosis factor alpha provide the thermogenic core for the wastage syndrome in cachexia.. Endocrinology.

[33] Anderson KA, Ribar TJ, Lin F, Noeldner PK, Green MF (2008). Hypothalamic CaMKK2 contributes to the regulation of energy balance.. Cell Metab.

[34] Schwartz MW, Porte D (2005). Diabetes, obesity, and the brain.. Science.

[35] Posey KA, Clegg DJ, Printz RL, Byun J, Morton GJ (2009). Hypothalamic proinflammatory lipid accumulation, inflammation, and insulin resistance in rats fed a high-fat diet.. Am J Physiol Endocrinol Metab.

[36] Bjornholm M, Munzberg H, Leshan RL, Villanueva EC, Bates SH (2007). Mice lacking inhibitory leptin receptor signals are lean with normal endocrine function.. J Clin Invest.

[37] Cotero VE, Routh VH (2009). Insulin blunts the response of glucose-excited neurons in the ventrolateral-ventromedial hypothalamic nucleus to decreased glucose.. Am J Physiol Endocrinol Metab.

[38] Figlewicz DP, Benoit SC (2009). Insulin, leptin, and food reward: update 2008.. Am J Physiol Regul Integr Comp Physiol.

[39] van den Pol AN (2003). Weighing the role of hypothalamic feeding neurotransmitters.. Neuron.

[40] Romanatto T, Roman EA, Arruda AP, Denis RG, Solon C (2009). Deletion of tumor necrosis factor-alpha receptor 1 (TNFR1) protects against diet-induced obesity by means of increased thermogenesis.. J Biol Chem.

