# Supplementary figures and images for: Unsaturated Fatty Acids Revert Diet-Induced Hypothalamic Inflammation in Obesity
Source: PLoS One. 2012 Jan 18;7(1):e30571. doi: 10.1371/journal.pone.0030571 (PMC3261210; doi:10.1371/journal.pone.0030571)

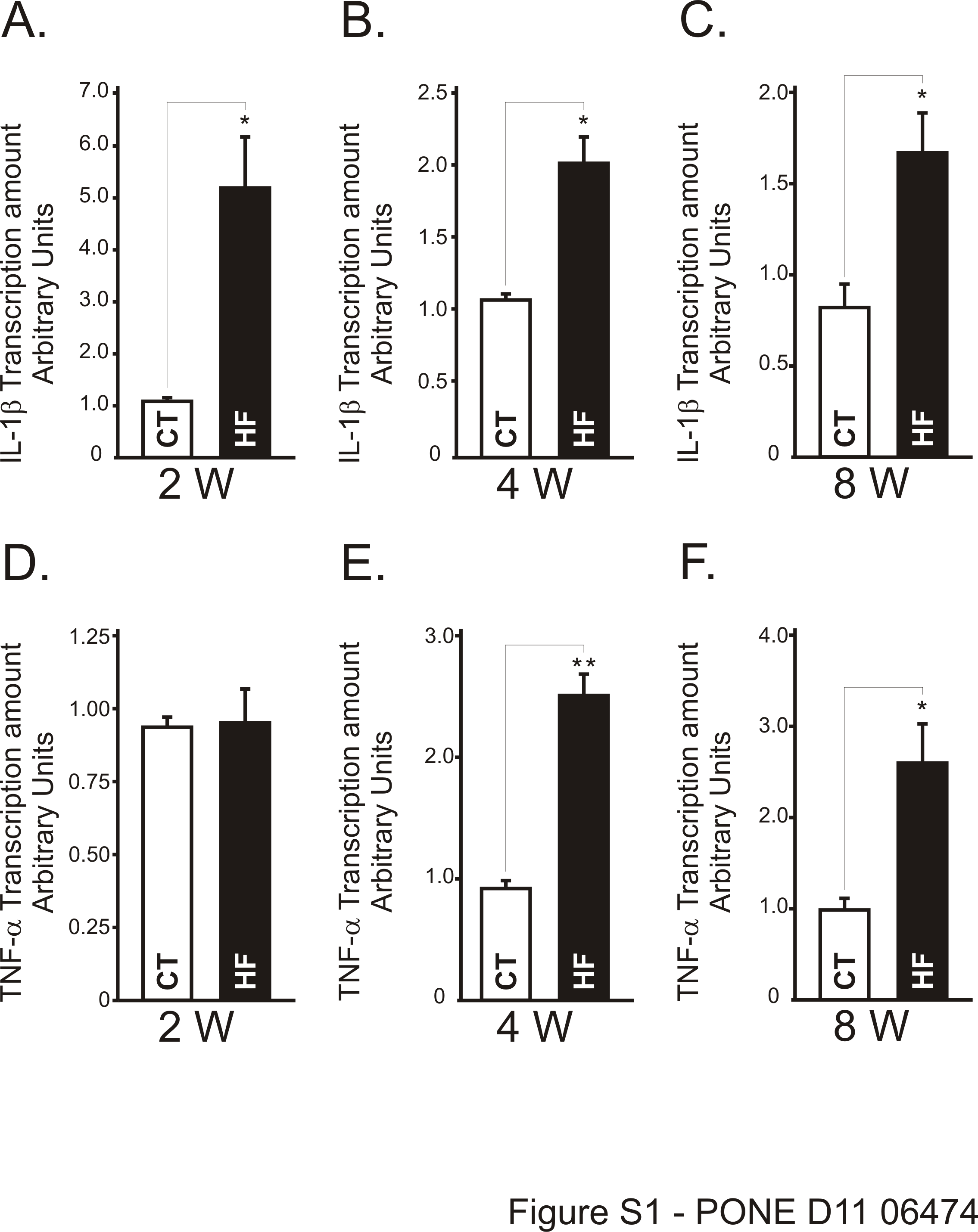

Supplement: Figure S1 — Time course of high-fat diet (HF)-induced expression of inflammatory markers in the hypothalamus. Swiss mice fed on chow (CT) or HF for 2, 4 or 8 weeks were employed for determination of the hypothalamic expression of interleukin-1b (A-C) or tumor necrosis factor-a (D-E) transcripts by real-time PCR. In all experiments, n = 5; *p<0.05 vs. CT. (TIF) [file pone.0030571.s001.tif]
